# Application of CMOS Technology to Silicon Photomultiplier Sensors

**DOI:** 10.3390/s17102204

**Published:** 2017-09-25

**Authors:** Nicola D’Ascenzo, Xi Zhang, Qingguo Xie

**Affiliations:** School of life science and technology, Huazhong University of Science and Technology, Wuhan 430073, China; dhmo@hust.edu.cn

**Keywords:** silicon photomultiplier, avalanche detection structures, geiger mode, low photon flux sensors

## Abstract

We use the 180 nm GLOBALFOUNDRIES (GF) BCDLite CMOS process for the production of a silicon photomultiplier prototype. We study the main characteristics of the developed sensor in comparison with commercial SiPMs obtained in custom technologies and other SiPMs developed with CMOS-compatible processes. We support our discussion with a transient modeling of the detection process of the silicon photomultiplier as well as with a series of static and dynamic experimental measurements in dark and illuminated environments.

## 1. Introduction

**Figure 1:**
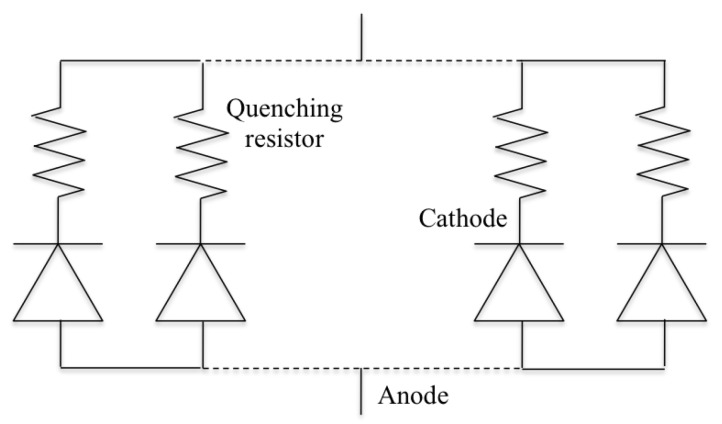
Schematics of a modern silicon photomultiplier (SiPM), composed of an array of single photon avalanche diode (SPAD)-like microcells with passive quenching.

The application of silicon technology to the development of low photon flux sensors—later known as silicon photomultipliers (SiPMs)—can be traced back to the late 1970s [1], when a space-distributed fine array of metal resistor semiconductor (MRS) micro-sensors was conceived with individual quenching and common output. Since then, the SiPM R&D is dominating the panorama of modern research in the field of low photon flux detectors, with the synergy and unique contribution of a large number of groups during the last 30 years [2,3,4,5,6,7,8,9,10]. Modern SiPMs are composed of an array of p/n-junctions (microcells) operated in Geiger mode, with individual passive quenching resistors, as reported in Figure 1. SiPM exhibits a clear response to single photons and an impressive photon number resolution at room temperature. In parallel, single photon avalanche diodes (SPADs) emerged in the area of single photon sensors [11]. The SPAD is a single photon detector operated in Geiger mode. Its optical sensing part is equivalent to a SiPM microcell. The layout of a SPAD differs from the one of a SiPM, as it also includes integrated active quenching electronic components [12]. In other words, the SiPM is equivalent to an array of SPAD-like microcells with passive quenching.

SiPM and SPAD substituted the photomultiplier tubes as low photon flux sensors in a large variety of applications, ranging from scintillator-based high energy physics to nuclear medicine equipment, radiation detectors, lidar systems in the automotive industry, and wearable devices, among others [13].

Recent advances in the conception of SiPM and SPAD are based on the investigation of the possibility of a full implementation of the photo-detector within standard CMOS, which enables the monolithic integration of read-out electronics and photo-detector on the same chip, with a significant reduction of power consumption and simplification of the operational conditions. Moreover, the use of standard CMOS technology facilities reduces the cost of the sensor, allowing an effective and stable mass production for industrial use.

The production of modern SiPM sensors within the standard CMOS process has a strong impact on the development of advanced integrated sensors for the detection of low photon flux and ionizing radiation. Further future implications are associated to the CMOS 3D-interconnection [14], which can improve the detection efficiency, as it was shown, by way of example, in the 130 nm Globalfoundries/Tezzaron [15] node. Within this technology, it is possible to develop new digital avalanche pixel structures, which provide a direct access to each pixel. The avalanche pixel image sensors and the avalanche pixel tracking sensors use this technology for low photon flux and ionizing radiation, respectively [16,17]. The application of CMOS SPAD sensor solutions to digital SiPM was demonstrated among others in the 800 nm [10] and 350 nm [18].

The rules imposed by the CMOS technology represent a limitation to the performances of the developed sensors. SiPM and SPAD may suffer localized breakdown conditions on the locally concentrated high electric field at the junction edges. The use of guard ring structures around the sensitive area of each microcell is mandatory in this respect in order to obtain a uniform electric field across the whole sensitive area. The CMOS technology offers few possibilities of implementing such guard rings [19,20,21,22]. By way of example, SPAD, SPAD arrays, and SiPM detection structures with several possible layout techniques for the implementation of guard rings were successfully implemented in 800 nm [10,23,24,25,26,27,28,29,30], 700 nm [31], 500 nm [32,33], 350 nm [18,34,35,36,37,38,39,40,41,42,43,44,45,46], 180 nm [47,48,49], 150 nm [50], 130 nm [15,51,52,53,54,55], and 90 nm [56,57] CMOS nodes, and were used to detect single photon signals on the basis of the avalanche breakdown process. Two main limitations of the CMOS technology remain; namely, the higher dark rate and the lower photon detection efficiency with respect to the custom-technology-based conventional SiPMs. As a possible solution, the mask set of CMOS processes is often enlarged with specific implantations, in order to allow the overlap between highly-doped and low-doped regions and to correct the doping profiles of the standard CMOS wells [58].

In this paper, we present a SiPM structure realized in 180 nm CMOS BCDLite technology at Globalfoundries. The CMOS process is used without any customization. We investigate the possibilities and the remaining limitations of this approach, as compared to SiPMs obtained both with custom-technology and in other standard CMOS nodes. This issue is supported by a SILVACO model of the detection structure and by a series of experimental results in dark and light conditions.

## 2. Materials and Methods

### 2.1. Simulation of SiPM

SiPM transient simulation is performed using the SILVACO suite [59]. We consider a p${}^{+}$/n-well diode structure composed of the typical doping concentrations offered by 180 nm CMOS technology. On a starting n-epitaxial layer with concentration $10^{15}$ cm${}^{-3}$, a gaussian shaped n-well is formed with maximal concentration $2.9\times 10^{17}$ cm${}^{-3}$ at a depth of 436 nm. The p${}^{+}$ implantation in the n-well has also a gaussian profile with maximal concentration $3.02\times 10^{20}$ cm${}^{-3}$ at a depth of 91 nm. The junction depth between p${}^{+}$ implantation and n-well is 142 nm.

The simulation includes the main physical processes involved in the SiPM operation. The electric field strength in the detection structure as a function of the total charge density distribution is governed by Poisson’s equation. When a microcell is biased in reverse mode, a depletion region is formed between the p- and n-doped regions. The thickness of the depletion region is approximately 1 μm. Within the depletion region, an electric field with maximal value of approximately $10^{5}$–$10^{6}$ V/cm is formed.

The continuity equation for the evaluation of the density of both carrier species includes generation and recombination mechanisms. In radiative recombination processes such as spontaneous or stimulated recombination, an electron is captured back in the valence band, with a consequent emission of respectively one or two photons. In non-radiative recombination processes, described by the Shockley-Read-Hall [60] and three-particle transition Auger models [61], the excess energy of the electrons is released respectively to phonons or to other carriers. In both radiative and non-radiative recombination processes, the excess energy can eventually be transferred to electrons, which are released in the conduction band. This process is naturally included in the recombination rates at equilibrium. The phonon excitation or the photons induced by recombination may in fact provide the necessary energy to an electron in the valence band to be released in the conduction band. Because of the properties of the Fermi distribution function of carriers in silicon, the process of electron-holes generation through recombination is temperature-dependent and is often called thermal generation of electron/hole pairs.

Although in normal conditions these processes would bring the junction back to the equilibrium of the doping concentrations, in the SiPM the produced electron/hole pairs are accelerated in the strong electric field and can trigger an avalanche multiplication chain due to the impact ionization process, which we include following the Selberherr’s model [62].

A more precise estimation of the involved processes requires the calculation of the carrier’s temperature, which we perform with the energy balance transport model. The thermal diffusivities of electrons and holes derive from the frictional interaction of carriers with lattice and among themselves. Carriers are accelerated by the electric field but lose momentum due to scattering processes, including phonon, carriers, and impurity scattering, surface and material imperfections. This effect is parameterized with a carrier mobility parameter, which is a function of electric field, lattice temperature, and doping concentration, and defines the carrier’s thermal diffusivity. In the low electric field regions of the detection structure, the relation between mobility and carrier concentration is included in the model through an experimental look-up table. When the electric field magnitude increases, the mobility depends on the strength of the electric field parallel to the current flow. Such dependence is parameterized using the Caughey and Thomas expression [63]. Relaxation times for energy losses due to the interaction of carriers with the lattice are also included, with characteristic relaxation times on the order of 1 ps. Finally, in heavy-doped regions, effects due to decreased band-gap separation are included according to [64].

The avalanche is self-sustaining, and a quenching mechanism is needed in order to recover to the first stability state of the SiPM. We opt for a passive quenching technique, using an external quenching resistor integrated in each SiPM microcell. The quenching resistor is included in the simulation as an external passive element. The current flow through the resistor causes a voltage drop on the junction, which reduces the actual junction bias to a value lower than the breakdown voltage. From the point of view of carriers kinematics modelling, the avalanche charge crowds within the depletion region and reduces the electric field, thus quenching the avalanche. The function of the large quenching resistor is indeed the limitation of the charge which can flow outside of the device. After the quenching occurs, the SiPM transits back to the initial state within a recovery time determined by the size of the junction capacitance and of the quenching resistor. Parasitic capacitance is not included in the model.

We calculate two variables in the simulation. The first is the current flowing in the circuit composed of the power supply, diode, and quenching resistor. The second is the voltage measured on a 50 Ω load resistor included in series after the diode and terminated to ground.

The micro-cell size is 50 μm × 50 μm. The computation is performed using adaptive space and time meshes, with a minimal required mesh size of 0.02 μm and time steps of $10^{-2}$ ps in order to achieve the required precision.

### 2.2. Fabrication of SiPM

The GLOBALFOUNDRIES (GF) 180 nm BCDLite process is an advanced mixed-signal CMOS process providing six metal layers, two polysilicon layers, high-resistivity polysilicon, and two types of transistor gates (3.3 V and 5 V). As with most of the available 180 nm standard CMOS processes at modern foundries, it does not offer an optical coupling (OPTO) module and a passivation layer also covers the sensitive region. This work is thus the basis for a discussion with other silicon facilities, which are always open to giving access to additional non-standard OPTO modules also at smaller scale. From a technological point of view, we also chose this process because it is used for standard electronics components (mainly for the automotive industry), and we aim at demonstrating that a prototype SiPM sensor can be obtained in full compatibility with such electronics. Although the process is also available also through multi-project wafer runs (which offer inexpensive prototyping), we opted for an engineering run, which has the advantage of a better customization.

**Figure 2:**
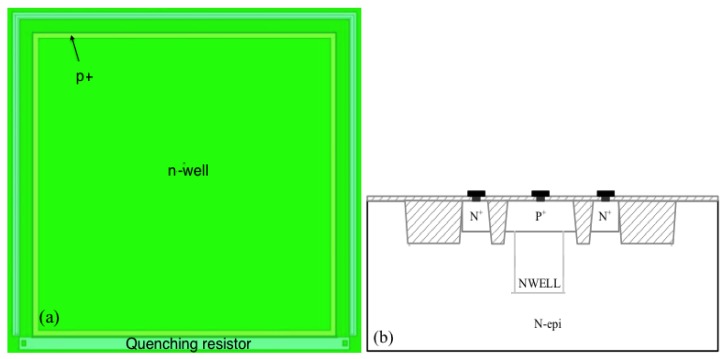
(**a**) Layout of a SiPM microcell in GLOBALFOUNDRIES (GF) 180 nm BCDLite process; and (**b**) cross-section of the microcell structure.

The photodetector structure is based on an n-epitaxial layer, on which the SiPM sensor is formed. Figure 2 shows the layout and the cross-section of one SiPM microcell implemented in the GF 180 nm BCDLite process. We note that in designing such a device, only standard masks provided by the technology are used. The microcell consists of a 50 μm×50 μm n${}^{+}$/p-well junction. GF does not share the exact doping of the wells with the customers.

A virtual guard ring p${}^{+}$/n-epi is formed on the periphery of the sensitive avalanche area in order to avoid the spontaneous triggering of breakdown avalanche in the high-gradient electric field on the periphery of the structure. Such a combination violates the inclusion rules of the p-well and n${}^{+}$ layers. This is the only needed violation of the standard design rules.

The shallow trench isolation (STI) surrounding the sensitive area of the avalanche pixel sensor is provided automatically by the CMOS technology rules. In our case, the STI contributes to the electric isolation of individual avalanche pixel sensor, allowing the necessary electric crosstalk protection of the SiPM microcells. The STI can be used functionally as a guard ring, physically removing the peripheral regions of the p${}^{+}$/n-junction [29,46]. Up to now, this option is under investigation, due to the problem of generation of additional leakage current [65].

In order to reduce the thickness of silicon oxide deposited on the active area of the sensor, we use only three metal layers out of the six available in the process.

As shown in the previous section, the avalanche breakdown process in the proposed p/n-junction structures is self-sustaining and requires a special quenching mechanism. In our design, the quenching element is passive. A 250 kΩ quenching resistor is implemented in the structure outside of the sensitive area on the basis of the high resistive polysilicon process. The resistor width is 0.8 μm, and its length approximately 50 μm.

On the wafer we produce single microcells test structures as well as SiPM sensor prototypes, consisting of an array of 20×20 microcells, connected in parallel, with common output. The SiPM prototype is designed in planar structure. The common cathode and anode are placed at the corners of the microcells array, as opening pads within the SiPM area.

## 3. Characterization Results

Two series of measurements have been carried out for the SiPM sensor prototype: a static and a dynamic characterization, including noise and light response studies. The benchmarking of the experimental results needs to follow a twofold approach. On the one side it is needed to benchmark against the mature available SiPMs, which are not always developed in standard CMOS technology but in dedicated lines with additional optimized masks. This first comparison allows us to understand how far the standard CMOS process is used in this work from the behaviour of specifically optimized processes for good-performing SiPM. On the other side it is needed to benchmark against other experimental attempts of using standard CMOS processes for the production of SiPM. This second comparison allows us to understand how the chosen CMOS technology process performs with respect to other available ones. A selection of representative examples is reported in Table 1.

### 3.1. Static Characterization

We start characterizing the single microcell by evaluating the static current–voltage characteristic in reverse bias mode of operation. The experiment is performed on the single microcell test structure at wafer level. A Keithley 2636 A source meter, connected to a computer, obtains measurements of current in reverse mode. This experiment is achieved by generating a sweep voltage between 0 V and 18 V, then measuring the current and limiting it to 20 μA in order to avoid damaging the device. The current versus voltage characteristics reveals the breakdown voltage and the dark current of the sensor.

**Figure 3:**
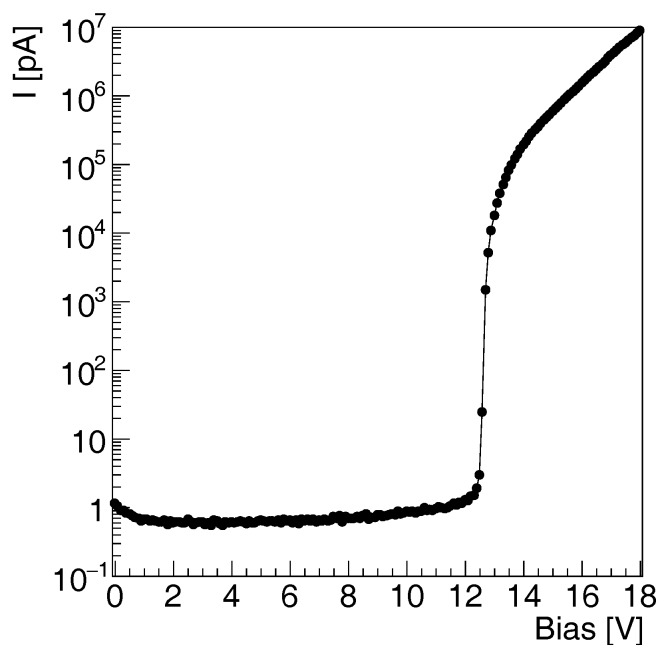
Current–voltage characterization of the SiPM microcell in dark condition.

Figure 3 shows the measured I-V characteristics of the microcell test structure in reverse mode. The structure exhibits a dark current below a few picoamperes before avalanche breakdown. At breakdown, the current rises abruptly up to a few microamperes. After breakdown, it gets limited by the quenching resistor and rises linearly. The breakdown voltage is approximately 12 V. The I-V curve of the SiPM prototype follows the results of the test microcell, without any deviation or unexpected behaviour. It is necessary to note here that the current measured in a static I-V curve represents the average of the current of a certain number of alternating dark pulses and quiescent state current levels within a 1 s time window.

The sharp rising edge of the breakdown in the I-V curve is also showing that there is no additional leakage current in the structure, which usually deteriorates the shape of the I-V curve [65]. In comparison with the custom-technology available SiPM, as shown on Table 1, the breakdown voltage obtained with the 180 nm GF BCDLite CMOS process is lower. This is because the technology lines specialized to the SiPM production introduce specific processes and additional masks in order to optimize the concentration of the wells forming the active area of the detector.

As reported in Table 1, the result is consistent with a similar SiPM obtained at the 180 nm CMOS technology node [49]. Breakdown voltages ranging from 10 V to 14 V are usually obtained at a scale lower than 180 nm, as in 90 nm [56,57] and 180 nm [20] CMOS nodes. This value depends on the doping of the standard CMOS wells, which range from $2\times 10^{17}$ to $5\times 10^{17}$ cm${}^{-3}$.

### 3.2. Dynamic Characterization

Based on the static characterization results, we performed a dynamic characterization of the SiPM prototype. A first series of measurements addresses the problem of dark rate in SiPM realized in CMOS technology framework. A second series of measurements is performed in light illumination condition.

#### 3.2.1. Noise Study

We measure the dark count rate by counting the number of avalanche signals produced in the SiPM sensor operated at the fixed bias voltage of 14 V, corresponding to 2 V above breakdown. The dark count rate is measured at room temperature and in dark conditions. The voltage amplitude of the signal of the SiPM is measured on a 50 Ω load resistor. The output voltage is connected to a fast amplifier, based on a two-stage voltage amplifier obtained with the GALI 5+ wide-band monolithic chip [66]. The total amplification gain is adjusted to 10 with a voltage divider between the two amplification stages. The signal is sent to a threshold discriminator (CAEN N844). The number of pulses above threshold registered within a 1 s observation time window.

**Figure 4:**
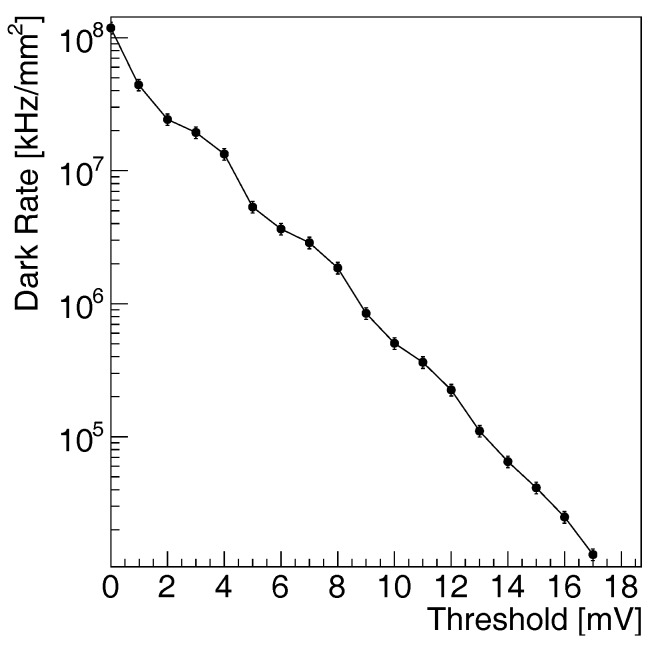
Dependence of the dark count rate of the SiPM prototype versus signal threshold at 14 V (2 V overvoltage) at 25${}^{\circ }$.

The measured dependence of the count rate versus signal amplitude is shown in Figure 4. The error bars represent the standard deviation of the measurement repeated on a set of 20 randomly selected chips produced on the same wafer dice. We observe a series of structures with decreasing amplitude. The first structure corresponds to the level of electronic noise, the second structure corresponds to one dark rate pulse, the *n*-th structure corresponds to n−1 coincident pulses from independent cells within the observation time window. The probability of measuring a large number of coincident pulses is increased by optical cross-talk, being the electric cross-talk suppressed by the STI. The dark rate at a threshold of 0.5 and 1.5 pulse amplitude is respectively $20\times 10^{6}$ kHz/mm${}^{2}$ and $10\times 10^{6}$ kHz/mm${}^{2}$. The optical cross-talk can be estimated from the ratio of the population of these levels as approximately 50%.

In comparison with the custom-technology SiPM benchmarks reported in Table 1, the obtained dark count rate is approximately 100 times larger. Although the operation over-voltage is within the value of the mature SiPM technology, the absolute bias voltage is much lower, as reported in the previous section. At such low breakdown voltage, the tunneling effect plays an important role and significantly deteriorates the dark count rate of the sensor. The degradation of the dark count rate in a standard CMOS process is a well-known problem. With reducing node scale, the wells concentration is increasing, with a consequent decrease of the breakdown voltage of any junction formed in the well. The design of a pn junction for SiPM sensors in modern CMOS technology faces the arising of tunneling effect, which is dominant for silicon at voltages lower than approximately 10 V [57]. This physics mechanism, together with the presence of STI and lower annealing temperature used at smaller scales, increases the amount of free electrons, which reach the conduction band spontaneously, deteriorating the noise performance of the SiPM sensor. The dark rate obtained in this study is compatible with the results at the 180 nm CMOS technology node [49].

Table 1 shows that SiPMs realized in CMOS technology generally exhibit a higher dark rate than SiPMs in custom-technology, due to either STI or higher doping concentration. An increase of the dark count rate was observed, for example, in the study at 350 nm and 90 nm CMOS technology node [35,57]. This is addressed in [58] as one of the main reasons for the introduction of additional masks in the fabrication of SiPM sensors on the basis of CMOS processes. The cross-talk is higher than in SiPMs with implemented optical trenches [67], suggesting that proper optical trenches need to be implemented for a SiPM with better performance.

#### 3.2.2. Light Study

We report here the measurement of packaged samples of the SiPM prototype in response to light. The experimental setup is composed of a fast LED with wavelength 550 nm. The amplitude of the driving pulse of the LED is adjusted with the programmable Keysight 81133A (Keysight, Santa Rosa, CA, USA) pulse pattern generator. A pulse width of 10 ns is chosen, approximately ten times less than the recovery time of the SiPM. The light pulses are delivered to the operation position in the light protected area by an optical fiber. The SiPM and the light source are kept at a relative distance of 1 cm. The optical table components provide an alignment precision of 0.1 mm between SiPM and optical source. The temperature of the experimental setup is controlled and kept constant at 25 ${}^{\circ }$C.

In order to calibrate the optical source, the C-Series SiPM by sensL, with a total area of 1 mm×1 mm is used in the same experimental setup. We detected a total of 80 photons. Considering that the photon detection efficiency of the used SiPM is approximately 25% at 550 nm, we estimate an average number of approximately 320 photons at the surface of the SiPM.

The voltage amplitude of the signal of the SiPM is measured on a 50 Ω load resistor connected to a fast amplifier, based on a two-stage voltage amplifier obtained with the GALI 5+ wide-band monolithic amplifier [66]. The total amplification gain is adjusted to 15 with a voltage divider between the two amplification stages. The charge of the signal is measured within an integration gate of 100 ns using the CAEN V1180 QDC in the VME Frame and stored in the control computer.

**Figure 5:**
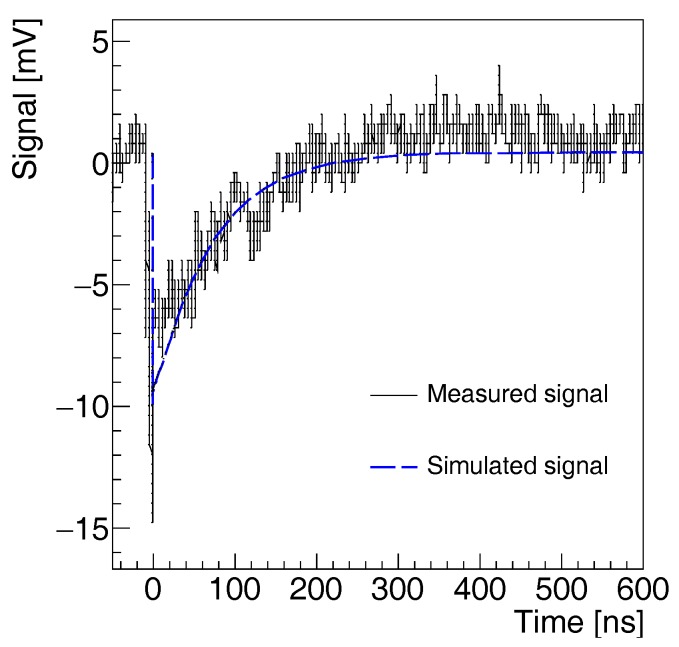
Simulated (blue dotted line) and measured (dark continuous line) signal of the SiPM prototype in response to a detected optical photon or a thermally generated electron/hole pair.

Figure 5 shows the measured signal corresponding to a single photon or to a thermally generated dark pulse. The signal exhibits a rise-time of approximately 120 ps and a characteristic recovery time of 67 ns. These timing properties are consistent with a quenching resistor of 250 kΩ and a capacitance of approximately 0.3 pF. The corresponding simulated signal—estimated on a 50 Ω load resistor, included in the simulation, and rescaled with the amplification stage used in the experiment—is shown in Figure 5 as a dotted blue line. The experimental observation is in agreement with the theoretical expectation. The impedance mismatch between diode, quenching resistor, and load resistor is responsible for the fast spike at the signal front in response to the fast quenching time. It is more pronounced in the data than in the simulation, because the parasitic capacitance is not included in the model.

**Figure 6:**
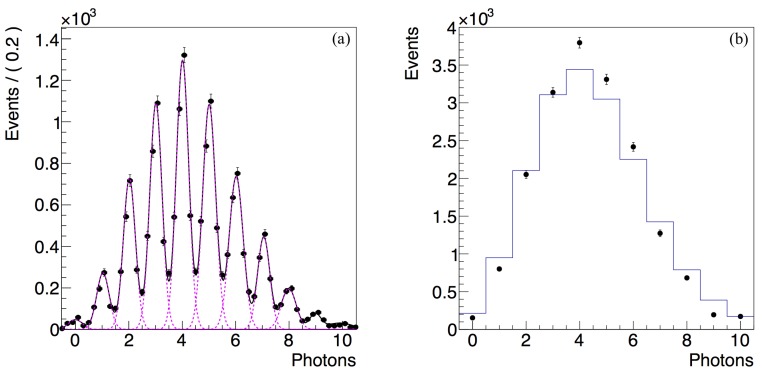
(**a**) Spectrum of the low photon flux detected by the SiPM prototype. The histogram is fit with a multi-gaussian fit (continuous line) with separately fitted areas (dotted line); (**b**) Statistical distributions of the number of events (area) of each peak in the spectrum and theoretical Poisson distribution.

Figure 6a shows the detected photon flux spectrum. On the *x* axis the measured charge is divided by the electron charge and the gain in order to represent the number of detected photons. The histogram consists of colorredhighly resolved peaks correspondeing to the number of detected photons. Each avalanche pixel detects one photon and provides as output the standard signal corresponding to a single photon. The common output of the SiPM is the analog sum of the signals from each avalanche pixel. In this condition, the first peak corresponds to 0 detected photons (electronic noise pedestal), the second one corresponds to 1 detected photon, the (n+1)th one to *n* detected photons.

The number of peaks contains the information about the number of detected photons. The separation between two successive peaks has a constant magnitude and corresponds to the total number of electrons produced in the avalanches process.

The analysis of the single photon spectrum is performed by fitting the spectrum with a multi-gaussian function composed of equally spaced gaussians. We follow the fitting technique reported in [68]. The fit result is shown in Figure 6a.

As a cross-check, we calculated the charge contained in the signal shown on Figure 5, and we verify that it is consistent with the average charge of the 1-photon peak in the spectrum in Figure 6a, which is measured as the fitted constant distance between the peaks. It corresponds to the internal amplification gain, and is estimated as $G=\left(1.406\pm 0.003\right)\times 10^{6}$ . It is consistent with the expected values obtained in previously designed devices, as reported in Table 1.

The resolution of the photon peaks is quantified using the fitted width $\sigma_{1}$ of the 1-photon-peak and its distance from the pedestal $\mu_{1}-\mu_{0}$:

(1) $$R=\frac{\sigma_{1}}{\mu_{1}-\mu_{0}}$$

In the proposed SiPM we obtain $R=\left(23.4\pm 0.1\right)$%. The photon peak resolution is determined by the electronic noise of the experimental setup and by the uniformity of the microcells. While the former aspect can be improved with dedicated low-noise readout electronics, the latter aspect is affected by two main intrinsic parameters of the CMOS technology process, namely the precision of the quenching resistor and the precision of the doping profiles in overlapping structures. It is useful to analyze here the contribution of these two features of the CMOS process.

The quenching resistor is obtained in the CMOS technology with a high resistance polysilicon (HRP). The doping stability of the HRP for different shapes and widths of the resistor is a common problem in CMOS silicon foundries. In 180 nm nodes and below, the precision of the resistor degrades with smaller length and width. The problem here is that the HRP is used in CMOS fabs mainly for digital electronic components, which do not need a high accuracy. The uniformity of the value of the resistor can have a spread of up to 10% in CMOS silicon foundries dedicated to digital electronics components. Analog applications as passive quenching in SiPM sensors require a high-quality HRP process, which is not always available at standard CMOS foundries.

**Figure 7:**
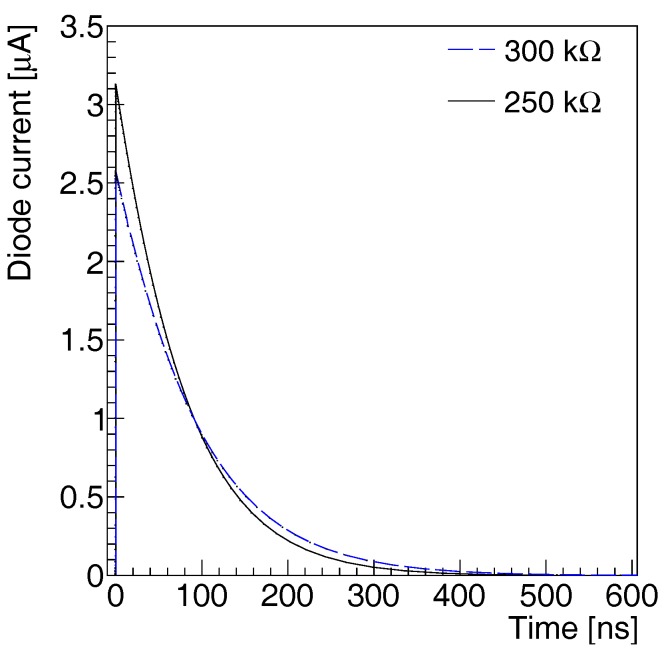
Simulated current of a SiPM sensor in response to a single photon or a thermally produced electron/hole pair for 250 kΩ (black continuous line) and 300 kΩ (blue dotted line) quenching resistor.

In order to study the effect of a change of the quenching resistor, we perform a transient simulation of the proposed structure using two sets of resistors, 250 kΩ and 300 kΩ. We report in Figure 7 the diode current corresponding to a single photon or to a thermally generated dark pulse. The current is flowing in the circuit composed of the diode, the bias source, and the quenching resistor. In both cases, the total charge contained in the signal is approximately $1.4\times 10^{6}$ electrons. The two signals have different peak amplitude, respectively 3 μA and 2.5 μA and decay time 78 ns and 93 ns. We conclude that a pixel-by-pixel non-uniformity of the quenching resistor does not affect the gain spread and the photon peak resolution, but has a negative impact on the overall time resolution of the detector.

Another significant technological issue when using the CMOS technology processes is the uniformity of the doping. In fact, the doping concentration of the wells affects the breakdown voltage. Power devices, logic, and analog electronic circuits do not require a precise breakdown voltage, as they operate at much lower biases. In these applications, the main optimization parameters to be considered are leakage current and electrical isolation, which should be under a specified tolerance threshold. The application of the standard CMOS processes to the fabrication of the SiPM sensor requires a much stronger constraint in terms of the needed tolerance of the doping profile.

Current implantation and annealing techniques guarantee a good uniformity of the well within the sensor. However, the problem here is at the edges of the junction, as the photodiode should be surrounded by a suitable guard ring (GR) to smooth the charge concentration and avoid the occurrence of local breakdown. Such guard rings are often implemented as regions with smaller doping levels (n-well structures or p-well implants) surrounding the active part of the sensor and lowering the electric field at the borders of the diode [29,46]. Implementing such GR structures at CMOS nodes smaller than 250 nm requires the violation of standard rules and may create non-uniformities in the obtained wells—in particular at the annealing stage—if the CMOS fabrication processes are used without any special consideration of this modified requirement.

We performed a transient simulation of a SiPM structure, modifying the doping concentration of the n-well. We observed that a change of 0.5% in the doping concentration contributes to a change of approximately 1% in the micro-cell gain. We conclude that a pixel-by-pixel non-uniformity of the doping concentration affects the gain spread and hence deteriorates the photon peak resolution.

As the photon peaks are well-visible and detected with a resolution of $\left(23.4\pm 0.1\right)$%, it is possible to conclude that although some standard rules are violated, the pixel-by-pixel non-uniformity is not dramatically affecting the gain spread in the design of the 50 μm side pixels at the Globalfoundries 180 nm CMOS node under investigation in this paper. This observation agrees with the results obtained, among others, at the Globalfoundries 130 nm CMOS node [15]. However, dedicated tests will be needed to experimentally verify the pixel-by-pixel non-uniformity in SiPMs with smaller or larger microcell size obtained at the same 180 nm technology node.

A second aspect of the spectrum in Figure 6a is that the area under each peak reflects the Poisson statistics of the photon detection in the SiPM structure and the contribution of the electronic noise. The average number of detected photons μ described by the Poisson distribution is calculated from the number of events of the pedestal peak $N_{0}$ and the total number of events *N*, applying the reconstruction formula under the hypothesis of a Poisson-distributed detected light:

(2) $$\mu =\log\frac{N}{N_{0}}$$

The average number of detected photons within the 100 ns time window is μ=4.34±0.01. Considering that, as stated above, the expected number of photons at the SiPM surface is approximately 320, we estimate a photon detection efficiency of approximately 1.3%. This result is below the expectation and is consistent with similar studies in standard CMOS technology processes. We observe in Table 1 that a photon detection efficiency lower than 4% at 420 nm due to the absence of an optical window and to the presence of a passivation layer are reported in the development of CMOS SiPM at the 350 nm HV AMS [41] and 180 nm [49] nodes.

Finally, it is necessary to state here that the result obtained using Formula (2) is only an approximation. In fact, as reported in the previous section, the sensor considered here exhibits a sizeable dark rate. It is hence expected that the response to the light source is affected by it. A statistical analysis of the single photon spectrum allows a better understanding of this phenomena. In Figure 6b we compare the theoretical expected Poisson distribution with the number of events (the area) of each peak in the experimental data obtained in the fit on Figure 6a. We observe a deviation between theory and experimental data, consisting approximately of a 15% excess of events at values higher than three photons. This shift in the observed distribution is due to dark rate and cross-talk, which cannot be neglected in this device. Correction schemes to this common problem in the SiPM application are reported in the literature [69,70].

## 4. Conclusions

We have presented an investigation concerning a silicon photomultiplier designed with the GF 180 nm BCDLite process. We found that with a minimal violation of the design rules (but still using only the standard production masks), it is possible to obtain a structure with a good photon signal resolution following the expectation of mature SiPM technology. However, the SiPM exhibits reduced photon detection efficiency due to the absence of an optical window in the GF 180 nm BCDLite process, high dark rate due to the tunneling effect at low breakdown voltage, and a relatively high cross-talk. We conclude that the addition of a dedicated OPTO process, of a mask for the tuning of the well doping in the sensor area and of optical isolation trenches is still needed in order to make a SiPM on one hand competitive with the mature SiPM technology and on the other hand fully compatible with the standard electronics developed in CMOS technology.

## Figures and Tables

**Table 1:** Comparison of the results obtained in this paper, using a 180 nm GF BCDLite CMOS process (in bold), with a selection of SiPMs obtained with standard CMOS technologies (upper part) and with custom-technology (lower part). The breakdown voltage (BV), gain, dark count rate and photon detection efficiency (PDE) at 410 nm are shown when available.

Techn.	Pixel	Typical	Gain	Dark Count	Cross-talk	PDE
Node	Size	Operation		@typ. bias		@410 nm
(nm)	(μm${}^{2}$)	(V)		(KHz/mm${}^{2}$)	(%)	(%)
800 [10]	2500	BV+1.5		$100\times 10^{5}$		15 (estimated)
800 [30]	2500	${\left(19.5\right)}_{BV}+3$		$3\times 10^{6}$		20
800 [30]	2500	${\left(17.5\right)}_{BV}+3$		$4\times 10^{5}$		25
800 [30]	2500	${\left(19.5\right)}_{BV}+2$		$3\times 10^{3}$		25
500 [32]	484	${\left(16.7\right)}_{BV}+1.5$		<120		15 (estimated)
350 [41]	2000	${\left(18.9\right)}_{BV}+1$		$2\times 10^{4}$	2.6	1.5
350 [44]	2500	${\left(25\right)}_{BV}+6$	$15\times 10^{6}$	503	33.5	34
350 [43]	913	${\left(26\right)}_{BV}+3$		$1.19\times 10^{3}$	2	
350 [18]	3185.47	${\left(27.5\right)}_{BV}+3$		75–100		
**180**	**2500**	${\left(12\right)}_{\mathrm{BV}}+2$	$1.4\times 10^{6}$	$20\times 10^{3}$	**40**	**1.3**
180 [49]	1024	${\left(10.3\right)}_{BV}+0.95$		$27\times 10^{3}$		4
130 [15]	324,900				
SensL [67]	100–2500	${\left(24.4\right)}_{BV}+2.5$	$4.3\times 10^{4}$	30–100	<2	40
Hamamatsu [9]	625–10,000	${\left(65\right)}_{BV}+2.6$	$1.26\times 10^{6}$	500		40

## Abbreviations

The following abbreviations are used in this manuscript:

BV	Breakdown Voltage
GF	GLOBALFOUNDRIES
GR	Guard Ring
HRP	High Resistance Polysilicon
MRS	Metal Resistor Semiconductor
OPTO	Optical coupling module
PDE	Photon Detection Efficiency
QDC	Charge Digital Converter
SiPM	Silicon Photomultiplier
SPAD	Single Photon Avalanche Diode
STI	Shallow Trench Isolation
